# Synthesis of Novel CuO Nanosheets and Their Non-Enzymatic Glucose Sensing Applications

**DOI:** 10.3390/s130607926

**Published:** 2013-06-20

**Authors:** Zafar Hussain Ibupoto, Kimleang Khun, Valerio Beni, Xianjie Liu, Magnus Willander

**Affiliations:** 1 Physical Electronics and Nanotechnology Division, Department of Science and Technology, Campus Norrköping, Linköping University, SE-601 74 Norrköping, Sweden; E-Mails: kimleang.khun@liu.se (K.K.); magnus.willander@liu.se (M.W.); 2 Biosensors and Bioelectronics Centre, Department of Physics, Chemistry & Biology (IFM), Linköping University, SE-581 83 Linköping, Sweden; E-Mail: valerio.beni@liu.se; 3 Department of Physics, Chemistry & Biology (IFM), Linköping University, SE-581 83 Linköping, Sweden; E-Mail: xjliu@ifm.liu.se

**Keywords:** CuO nanosheets, hydrothermal growth method, non-enzymatic glucose sensor, selectivity, reproducibility

## Abstract

In this study, we have developed a sensitive and selective glucose sensor using novel CuO nanosheets which were grown on a gold coated glass substrate by a low temperature growth method. X-ray differaction (XRD) and scanning electron microscopy (SEM) techniques were used for the structural characterization of CuO nanostructures. CuO nanosheets are highly dense, uniform, and exhibited good crystalline array structure. X-ray photoelectron spectroscopy (XPS) technique was applied for the study of chemical composition of CuO nanosheets and the obtained information demonstrated pure phase CuO nanosheets. The novel CuO nanosheets were employed for the development of a sensitive and selective non-enzymatic glucose sensor. The measured sensitivity and a correlation coefficient are in order 5.20 × 10^2^ μA/mMcm^2^ and 0.998, respectively. The proposed sensor is associated with several advantages such as low cost, simplicity, high stability, reproducibility and selectivity for the quick detection of glucose.

## 1. Introduction

Cupric oxide (CuO) exhibits a narrow band gap of 1.2 eV and has been widely investigated for various potential applications such as lithium ion electrodes, sensors, high critical temperature super- conductors, field emission emitter catalysts, *etc.* [1–9]. The existence of nanodimensional CuO is accompanied by several advantages including high theoretic capacity of 670 mAh^−1^, non-toxicity, cheapness and facile preparation in different morphologies [10]. The electrochemical performance of CuO nanostructures is highly dependent on the morphology and size [11,12]. Therefore, in the recent past extensive efforts have been put forward to address the size and shape control of CuO nanomaterials for the improvement in the cyclability of CuO-based electrodes. Recently, CuO nanomaterials have been produced in a variety of morphologies such as nanoparticles, nanoneedles, nanowhiskers, nanowires, nanoshuttles, nanorods, nanotubes, nanoleaves, and nanoribbons by using the solution-based approach and vapor phase growth techniques [13–21]. CuO can also be obtained in different complex nanostructures such as nanoellipsoids [22], peanut-like nanostructures [23], nano-dendrites [24], prickly/layered microspheres [25], and dandelion-like hollow morphology [26]. The solution-based growth approach is popular among the scientific community due to its several attractive features such as highly promising growth approach, low cost, and high yields of the to be prepared nanomaterials. The growth parameters such as concentration of reactants, temperature, time and pH of growth solution all have significant effects on the control of morphology and size of synthesized nanostructures. However, among these parameters pH is the one parameter which can be used for the tuning of CuO morphology, thus various nanostructures of CuO can be obtained by varying the pH of the growth solutions [11,27]. The hydrothermal growth method is known as environmentally friendly and the CuO nanomaterial can be obtained at low temperature with controlled morphology and size. Various glucose sensors have been fabricated by exploiting the attractive properties of nanomaterials such as Au [28,29], Pt [30–32], Pt-Pb [33,34], Cu [35] nanoparticles, Pt nanotubes [36], Au [37] porous films, Au [38] nanowires, *etc.* The enzyme-free detection of glucose is becoming more popular in order to overcome the disadvantages of glucose sensors based on immobilized glucose oxidase. The enzyme-free detection of glucose is associated with several advantages such as fabrication and storage conditions that are cheap and simple, no enzyme denaturation and degradation, and the sensors have more stability towards higher temperatures, pH and more resistance to toxic chemicals which could reduce the working performance of enzyme-based glucose sensors [39,40]. Non-enzymatic glucose sensors have been fabricated using different nanomaterials, including metals and metal oxides such as Pt [30], Cu_2_O [41], RuO_2_ [42], NiO [43–45], Co_3_O_4_ [46], MnO_2_ [47] and CuO nanoparticle-modified carbon nanotube electrodes [48].

Among these nanomaterials, copper and copper oxide-based electrodes for the sensing of glucose are largely used because of the direct electro-oxidation of glucose on the copper and CuO which is further increased by the multi electron oxidation by the surface oxide layer [49]. The copper and CuO nanomaterials are cost effective, non-toxic in some cases, and simple to synthesize. They possess high specific capacitance, and hence are considered suitable materials for the sensing of glucose. The morphology has a large impact on the catalytic properties of copper and CuO nanomaterials, Compton *et al.* have developed non-enzymatic glucose sensors for carbohydrates and hydrogen peroxide using two morphologies of CuO including microparticles and nanorod bundles and observed that CuO nanorod bundles showed better catalytic performance for the oxidation of glucose than the microparticles of CuO [50]. Recently, several morphologies of copper and CuO have been synthesized and potentially applied for the electrochemical and catalytic purposes [51], however morphologies with enhanced catalytic performance are still needed.

In the present work, a novel CuO morphology was synthesized in a highly alkaline medium and the resulting novel CuO nanostructures were used for the development of an enzyme-free glucose sensor. The non-enzymatic glucose sensor based on these novel CuO nanosheets is highly sensitive, selective, stable and reproducible.

## 2. Experimental Section

Copper nitrate hemipentahydrate, hexamethylenetetramine, D-glucose, ascorbic acid, uric acid, fructose, dopamine, ammonia solution, nitric acid, were purchased from Sigma Aldrich (Stockholm, Sweden). All other chemicals used were of analytical grade.

The gold layer on the glass substrate was deposited according to our reported work [52]. CuO nanostructures were grown on the gold layer-coated glass substrate by a hydrothermal growth technique and the growth process was as follows: the gold coated glass substrate was washed with isopropanol in an ultrasonic bath for 10 min and cleaned with deionized water and subsequently dried with a flow of air at room temperature. Then a seed layer of copper acetate monohydrate was deposited on the cleaned substrates by the spin coating technique and annealed at 120 °C for 10 to 20 min. The growth solution was prepared by mixing equimolar concentrations of copper nitrate hemipentahydrate and hexamethylenetetramine in 100 mL of the deionized water. The annealed substrates containing seed particles were affixed in a Teflon sample holder and vertically dipped in the growth solution at 80 °C for 4–6 h in the preheated oven. The pH 11 of growth solution was adjusted by using 0.1 M nitric acid and 25% ammonia solution.

The structural characterization was carried using scanning electron microscopy and X-ray differaction techniques. The composition of the CuO nanomaterial was studied by the XPS technique. For the measurement of electrochemical responses, CuO nanostructures were used as working electrode, platinum wire as counter electrode and silver-silver chloride as reference electrode by using the potentiostat. All the glucose sensing experiments were performed at room temperature and in alkaline 0.10 M NaOH solution.

## 3. Results and Discussion

### 3.1. The Structural Characterization of Grown CuO Nanomaterial

**Figure 1.**
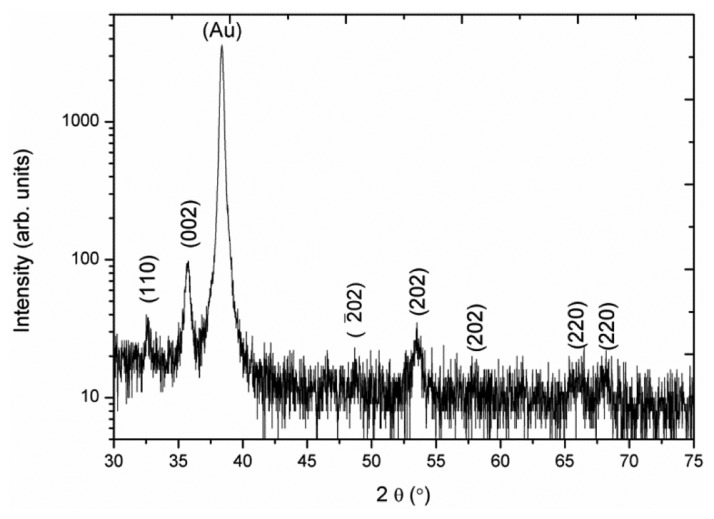
The XRD pattern of CuO nanosheets.

Figure 1 shows the XRD patterns of a CuO sample prepared by the hydrothermal growth method. The synthesized CuO nanosheets are purely composed of monoclinic crystalline phase and the obtained diffraction peaks are in accordance with the reported standard JCPDS card no: (80-1917). Meanwhile no other diffraction peaks for impurities are detected, which confirms the high purity of the as-synthesized CuO nanosheets in the present study.

**Figure 2.**
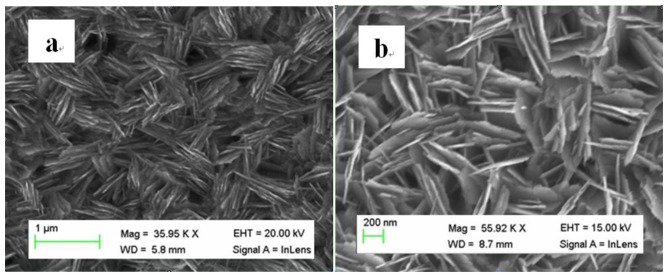
The SEM image of CuO nanosheets (**a**) low magnification image; and (**b**) high magnification image.

Scanning electron microscopy was used for the study of the morphology of the prepared CuO nanostructures. Figure 2(a) shows the typical low magnification SEM image for the synthesized CuO. It can be inferred that the nanostructures appeared like as interconnected nanosheets having an average thickness of 10–20 nm. The high magnification SEM image clearly shows the interconnection of several nanosheets of CuO as shown in Figure 2(b). The CuO nanostructures obtained in the growth solution of pH 11 could be due to higher concentration of hydroxide ions in the growth solution which improved the speed of the formation process of nanosheets by providing plenty of growth nuclei for the CuO nanomaterial.

In the beginning of growth process, a few CuO nuclei were produced and the surface of these nuclei is either negatively or positively charged, thus the oppositely charged species such as OH^−^ or Cu^2+^ will be attracted by the surface nuclei. This process of aggregation of opposite charges ends with the formation of CuO nanomaterial. The obtained information indicates that NH_3_. H_2_O has a significant influence on the morphology, number of nuclei of CuO and the connectivity of various nanosheets.

**Figure 3.**
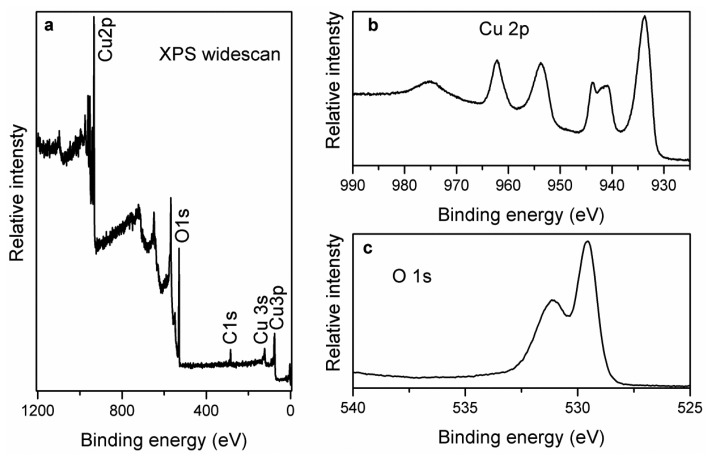
The XPS study of CuO nanosheets.

The chemical composition of CuO nanosheets was studied by the XPS technique. Figure 3(a) shows the combined XPS spectrum for the elements present in the prepared sample and it can be inferred those three distinct peaks at 284.00 and 531.00 eV are observed for C 1s, and O 1s respectively [53]. Moreover, the measured peaks at 933.30, 121.10 and 77.00 eV could be assigned to the Cu 2p, Cu 3s and Cu 3p respectively [54]. Figure 3(b,c) shows the XPS spectra of O 1s and Cu 2p respectively. In the O 1 s spectrum, two peaks have been observed which can be assigned to the O^2-^ in CuO at 529.47 eV and the peak at 531.15 eV is correlated to the adsorbed oxygen respectively. For Cu 2p, the measured peak at 933.60 eV corresponds to the binding energy of Cu 2p3/2 which is matched to the reported works [55] as shown in Figure 3(c). In addition to this, two shake up peaks demonstrate the formation of CuO compound on the gold coated glass substrate by the hydrothermal method as shown in Figure 3(c). The obtained XPS information indicates the purity of the CuO phase, and is consistent with the XRD data.

### 3.2. The Cyclic Voltammetry Study of the CuO Nanosheets and Amperometric Response

**Figure 4.**
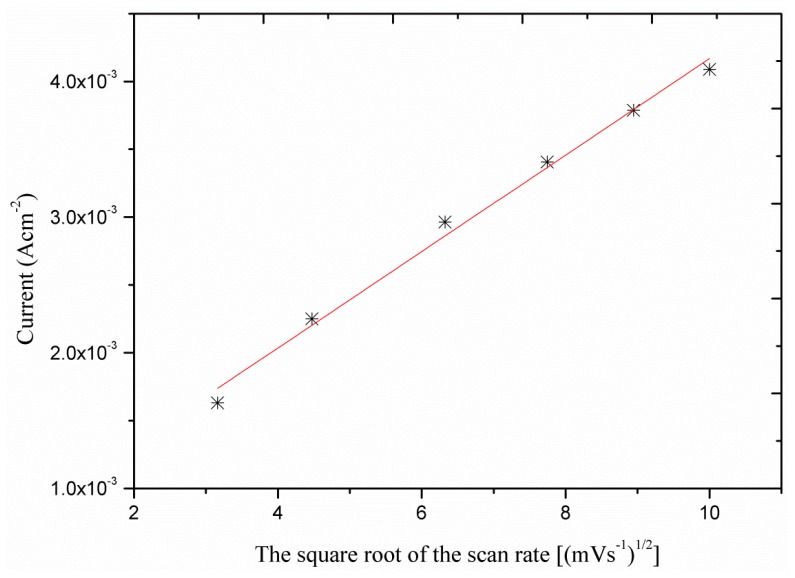
Calibration curve of the current *versus* different scan rate measured in 1.96 mM glucose concentration.

In order to obtain a better understanding of the oxidation process the voltametric response (CVs) as a function of the scan rate was investigated. As it can be seen the sensor electrode showed linear response with the square root of the scan rates (Figure 4) which indicates that the process is diffusion dependent.

**Figure 5.**
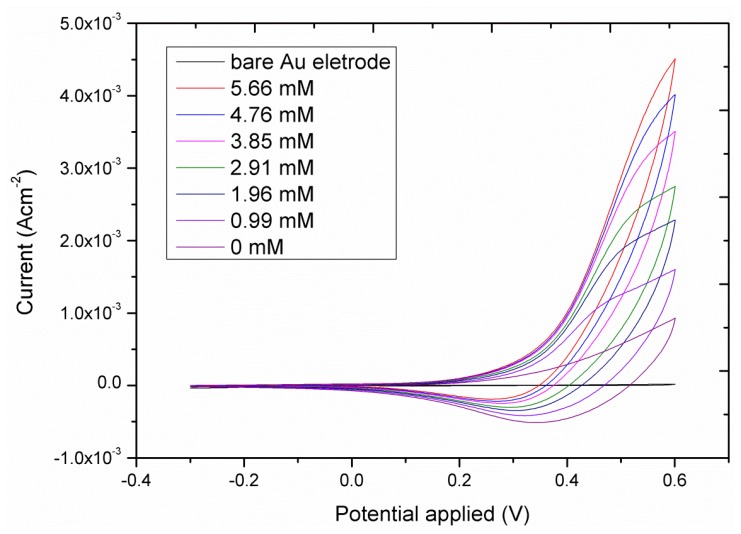
The CVs curve of CuO nanosheets based electrode in different concentrations of glucose at the scan rate of 10.00 mV/s.

**Figure 6.**
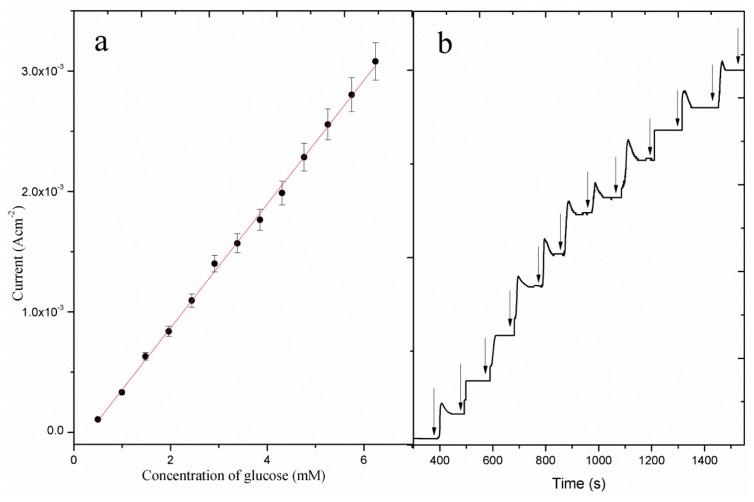
(**a**) The calibration curve of the current *versus* glucose concentrations by amperometric measurement at applied potential of 0.50 V; (**b**) The response time curve of the proposed glucose sensor at applied potential of 0.50 V with successive addition of glucose in 0.10 M NaOH solution.

The cyclic voltametric response of the CuO nanosheet-based electrode for different glucose concentrations is shown in Figure 5. As it can be seen from this figure, the addition of increasing concentrations of glucose in the electrolyte solution (0.10 M NaOH), made the anodic peak current increase, which illustrates the fast oxidation of glucose using the enhanced catalytic properties of the CuO nanosheets. The amperometric detection of glucose oxidation was studied at the applied potential of 0.50 V for different concentrations of glucose and the observed response is shown in Figure 6(b). It can be observed that the amperometric response was found to be linear for the 5.00 × 10^−1^-1.00 × 10^1^ mM glucose concentrations. The calibration curve of the proposed CuO nanosheets based sensor is shown in Figure 6(a) and the measured sensitivity and a correlation coefficient are in order of 5.20 × 10^2^ μAmM^−1^cm^−2^ and 0.998, respectively. The most noticeable thing in the present sensor is the higher sensitivity of its amperometric response than of the voltametric response, probably due to a reduction of the background response. Moreover, higher current is generated during the amperometric measurement which indicates the possible quick oxidation of glucose on the sheet-like morphology of CuO. Moreover, this higher sensitivity could be due to the high electrocatalytic properties exhibited by CuO nanosheets. As it can be seen from Figure 6(b), a fast response was recorded with a steady current signal being achieved within 10 s of the addition of the glucose.

### 3.3. Reproducibility and Stability Study of the CuO Nanosheet-Based Non-Enzymatic Glucose Sensor

Eight independent CuO nanosheet-based sensor electrodes were fabricated on a gold coated glass substrate in an alkaline pH of 11. The current response of these electrodes was measured for 0.10 mM glucose at 0.50 V. The relative standard deviation was found to be 4.30%, which indicates acceptable reproducibility of the developed non-enzymatic glucose sensor using CuO nanosheets. The long term stability of the sensor was evaluated for a period of three weeks by measuring the current response for glucose. The catalytic current of the proposed sensor was monitored on alternative days and the sensor maintained 90% of its original sensitivity after three weeks, which indicates the long term storage and working stability of the CuO nanosheet-based glucose sensor.

**Figure 7.**
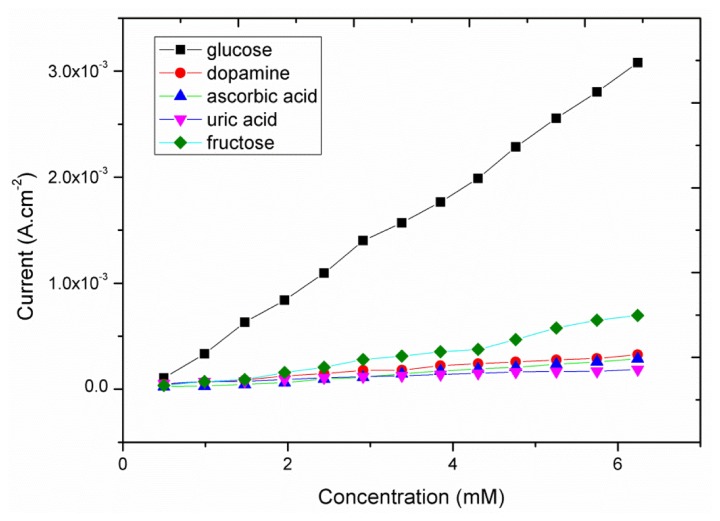
The calibration curve of the proposed glucose sensor in the presence of common interferents.

The natural selectivity for the non-enzymatic glucose sensor is a very important factor for the performance evaluation because easily oxidizable substances such as ascorbic acid, dopamine, uric acid and fructose are well known interfering agents during glucose measurements. The glucose concentration in the human blood is *ca.* 30 times higher than that of the common interferents; interference studies were performed by comparing the amperometric response of the glucose to those of the interfering molecules (Figure 7). As it can be seen from this Figure in the presence of increasing concentrations of glucose a significant increase in the oxidation response was obtained; on the other end only a limited current variation was recorded in the case of the interfering molecules. Thus the observed behavior of the proposed glucose sensor using the CuO nanosheets showed a natural selective response for the detection of glucose in the presence of these common interferents. The obtained results of the presented glucose sensor are comparable with those reported other enzyme-free glucose sensors (Table 1).

## 4. Conclusion

Novel CuO nanosheets were fabricated on a gold coated glass substrate by a hydrothermal method. The structural study of the novel CuO nanosheets was carried out using the XRD and SEM techniques. The chemical composition of the novel CuO morphology was studied by the XPS technique. The structural and composition studies have revealed that the CuO nanosheets are highly dense, uniform and exhibit a good crystalline array. Furthermore, these novel CuO nanosheets were used for the development of a sensitive non-enzymatic glucose sensor. The sensor possesses high sensitivity of 5.20 × 10^2^ μAmM^−1^·cm^−2^, a wide glucose detection range, good selectivity, reproducibility and stability. The proposed CuO nanosheet-based non-enzymatic glucose sensor may have potential for the analysis of glucose in real samples.

## Figures and Tables

**Table 1.** Comparison of the present CuO nanosheets sensor electrode with other non-enzymatic glucose sensors.

**No.**	**Modification on GCE**	**Electrochemical Technique**	**Sensitivity (μAmM^−1^cm^−2^)**	**Linear Range (mM)**	**Detection Limit (mM)**	**Reference**
1	RGO-Ni(OH)_2_	Chronoamperometry	1.14 × 10^1^	2.00 × 10^−3^ - 3.10 × 10°	6.00 × 10^−4^	[56]
2	Ni(OH)_2_-Graphene	Chronoamperometry	4.94 × 10^2^ 3.28 × 10^2^	1.00 × 10^−3^ − 1.00 × 10^−2^ 1.00 × 10^−2^ − 1.00 × 10°	6.00 × 10^−4^	[57]
3	Nickel nanospheres-RGO	Chronoamperometry	8.13 × 10^2^ 9.37 × 10^2^	1.00 × 10^−3^ − 1.10 × 10^−1^ 1.00 × 10^−3^ − 1.00 × 10^−2^	–	[58]
4	DNA dispersed Graphene-NiO	Chronoamperometry	9.00 × 10° 1.43 × 10^1^	1.00 × 10^−3^ − 8.00 × 10° 1.00 × 10^−3^ − 2.00 × 10°	2.50 ×1 0^−3^	[59]
5	CNT with Bimetallic Pt-M (M = Ru and Sn)	Chronoamperometry	8.10 × 10^−1^ 8.10 × 10^−1^	5.00 × 10° − 1.00 × 10^2^ 3.00 × 10° − 1.00 × 10^2^	5.00 × 10° 3.00 × 10°	[60]
6	CuO flower and nanorods	Chronoamperometry	7.10 × 10^2^ 3.71 × 10^2^	4.00 × 10^−3^ − 8.00 × 10°	4.00 × 10^−3^	[2]
7	CuO nanosheets	Chronoamperometry	5.20 × 10^2^	5.00 × 10^−1^ − 1.00 × 10^1^	1.00 × 10^−4^	Present work
